# Aryl Hydrocarbon Receptor and Kynurenine: Recent Advances in Autoimmune Disease Research

**DOI:** 10.3389/fimmu.2014.00551

**Published:** 2014-10-29

**Authors:** Nam Trung Nguyen, Taisuke Nakahama, Duc Hoang Le, Le Van Son, Ha Hoang Chu, Tadamitsu Kishimoto

**Affiliations:** ^1^Laboratory of Immune Regulation, WPI-Immunology Frontier Research Center, Osaka University, Suita, Japan; ^2^National Key Laboratory of Gene Technology, Institute of Biotechnology, Vietnam Academy of Science and Technology, Hanoi, Vietnam; ^3^Department of RNA Biology and Neuroscience, Graduate School of Medicine Osaka University, Suita, Japan

**Keywords:** dioxin receptor, indoleamine 2,3-dioxygenase, transcription factor, autoimmunity, immune regulation

## Abstract

Aryl hydrocarbon receptor (AHR) is thought to be a crucial factor in the regulation of immune responses. Many AHR-mediated immunoregulatory mechanisms have been discovered, and this knowledge may enhance our understanding of the molecular pathogenesis of autoimmune inflammatory syndromes such as collagen-induced arthritis, experimental autoimmune encephalomyelitis, and experimental colitis. Recent findings have elucidated the critical link between AHR and indoleamine 2,3-dioxygenase (IDO) in the development of regulatory T cells and Th17 cells, which are key factors in a variety of human autoimmune diseases. Induction of IDO and IDO-mediated tryptophan catabolism, together with its downstream products such as kynurenine, is an important immunoregulatory mechanism underlying immunosuppression, tolerance, and immunity. Recent studies revealed that induction of IDO depends on AHR expression. This review summarizes the most current findings regarding the functions of AHR and IDO in immune cells as they relate to the pathogenesis of autoimmune diseases in response to various stimuli. We also discuss the potential link between AHR and IDO/tryptophan metabolites, and the involvement of several novel related factors (such as microRNA) in the development of autoimmune diseases. These novel factors represent potential therapeutic targets for the treatment of autoimmune disorders.

## Roles of AHR in the Immune System

Aryl hydrocarbon receptor (AHR) is a ligand-activated member of the Per–Arnt–Sim (PAS) family of basic helix–loop–helix (HLH) transcription factors. AHR mediates cellular responses to toxins or its ligands, including TCDD, 6-formylindolo[3,2-b]carbazole (FICZ), kynurenine, and 2-(1′H-indole-3′-carbonyl)-thiazole-4-carboxylic acid methyl ester (ITE) (1–4). AHR forms an active complex in the cytoplasm with chaperone proteins such as heat shock protein 90 (HSP90), AHR-interacting protein (AIP), and p23 (5–7). Once bound to its ligands, the AHR complex translocates to the nucleus and binds AHR nuclear translocator (Arnt). The resultant AHR–Arnt heterodimers bind specific motifs, called dioxin-responsive elements (DREs), in the promoter region of target genes. These targets, the so-called AHR battery genes, include *CYP1A1*, *CYP1A2*, *CYP1B1*, and other members of cytochrome P450 family (8–11). Several pathways are involved in the regulation of AHR, including proteasomal degradation of AHR, ligand metabolism by CYP1A1, and formation of the AHR–Arnt complex (12, 13). One of these pathways involves an inhibitory peptide. Mimura et al. isolated a cDNA clone that encode a polypeptide with high similarity to the sequence of the bHLH/PAS of AHR (14). This polypeptide can repress the transcriptional activity of AHR by competing with AHR for binding to Arnt and by binding to the enhancer sequence XRE, upstream of the AHRR gene; therefore, this peptide is designated AHR repressor or AHRR. Expression of AHRR is induced by the AHR/Arnt heterodimer through binding to XRE, resulting in feedback inhibition of AHR. In addition, several transcription factors can interact and regulate AHR signaling; these include STAT-1, STAT3, STAT5, Pai-2, Sp1, c-maf, and Bach2 in certain cell types (15–24). AHR is activated in many immune cell types, including T cells, B cells, NK cells, macrophages, and dendritic cells (DCs), as well as in epithelial cells, Langerhans cells, innate lymphoid cells, intraepithelial lymphocytes, and microglia (15, 16, 20, 21, 25–40). Depending on the presence of specific ligands, AHR activation may suppress or exacerbate experimental autoimmune diseases. For examples, TCDD and ITE can suppress experimental autoimmune encephalomyelitis (EAE), a model of multiple sclerosis (MS), whereas FICZ exacerbates disease development (17, 41–43). Differences in the stability and structure of these ligands, as well as their affinity for AHR, should be taken into account when considering their mode of action in the activation of AHR. In addition, AHR seems to be regulated by unraveled factors such as transcription factors, tryptophan metabolites, feedback regulation of the cytokine network, and microRNA (miR). Below, we will discuss in detail the factors that may interact with AHR to modulate immune responses.

## IDO and Tryptophan Metabolites

Indoleamine 2,3-dioxygenase (IDO) is the rate-limiting enzyme in extrahepatic catabolism of the essential amino acid tryptophan via the kynurenine pathway (44). IDO is constitutively expressed in placenta, but is also highly expressed in epididymis, gut, lymph nodes, spleen, thymus, and lung (45). IDO converts tryptophan into kynurenine and other downstream metabolites, some with neuroactive properties, such as kynurenic acid, 3-hydroxykynurenine, quinolinic acid (QUIN), and serotonin. Deficiency or overexpression of IDO can result in changes in the levels of these neuroactive IDO-mediated metabolites, ultimately leading to neuronal disorders. For instance, elevated levels of QUIN, a potently neurotoxic *N*-methyl-*D*-aspartate (NMDA) receptor agonist, can cause not only neurodegenerative conditions such as Huntington’s disease (46) and Parkinson’s disease (47), but also infections of the central nervous system and psychiatric diseases such as depression (48). On the other hand, in pregnant mice, IDO may play a role in preventing the rejection of allogeneic fetuses (49). In addition, blocking IDO activity with the competitive inhibitor 1-methyl tryptophan (1-MT) selectively disrupts the maintenance of pregnancy in mice (49, 50). Consistent with the observations that IDO induces tolerance in allogeneic fetuses, IDO expression, especially in DCs, suppresses the T cell response (49, 51, 52). Various mediators can induce IDO expression and activity, including IFN-γ, TNF-α, IL-1β, and IL-6 (53–58). Recent work showed that the induction of IDO depends on AHR (31, 59, 60). Furthermore, kynurenine has been identified as a potent AHR agonist (2, 61, 62).

Immunoregulatory IDO activity and tryptophan metabolites participate in the regulation of many cell types, including T cells, DCs, monocytes, macrophages, and microglia, that play specific roles in immune responses and regulate the development of immune-mediated inflammatory diseases (62–66). Alberati-Giani et al. showed that IFN-γ induced the expression of IDO activity in immortalized murine macrophages (MT2) and microglial (N11) cells. IFN-γ-treated MT2 cells, but not N11 cells, were able to produce detectable amounts of radiolabeled 3-hydroxyanthranilic and QUIN from L-[5-^3^H] tryptophan. In addition, Heyes et al. demonstrated that increased activities of IDO, kynurenine-3-hydroxylase, and kynureninase in infiltrating primary macrophages may accelerate the synthesis of QUIN, L-kynurenine, and kynurenic acid in conditions of brain inflammation (48). Taken together, these findings suggested that infiltrating macrophages may contribute high amount of cerebral QUIN in brain inflammation (48, 67). Besides, IDO has been demonstrated to be constitutively expressed in DCs (68). Particularly, CD8α^+^DC exhibited high functional activity of IDO while CD8^−^ fraction of DC did not exhibit significant enzyme activity (68). Moreover, it has been shown that the expression of IDO makes CD8^+^DC treated with IFN-γ capable of affecting apoptosis of T helper type 1 (Th1) cells (68, 69). Fallarino et al. also found that tryptophan metabolites such as 3-hydroxyanthranilic and QUIN induce the selective apoptosis *in vitro* of murine Th1 cells at relatively low concentrations of kynurenines (70). Together, IDO and kynurenines metabolites are able to be induced in various cell types under different stimuli regulating many immune responses. Modulation of IDO activity and/or kynurenine pathway may develop therapeutics for inflammatory diseases. In addition to IDO, tryptophan 2,3-dioxygenase (TDO) originated from liver and neuron, is also an important rate-limiting enzyme in the tryptophan metabolism. TDO plays a pivotal role in tumor development and various diseases in the brain (61, 71–73).

The transcriptional regulation of IDO is mediated by two main pathways, IFN-γ-dependent and -independent, which involve transcription factors such as NF-κB, STAT-1, and IRF-1 (74). We will discuss the translational regulation of IDO by AHR in the following section.

## Translational Regulation of IDO

Although the regulatory roles of IDO in tryptophan metabolism in immune regulation have been extensively studied, the mechanisms by which IDO is controlled at the pre-, co-, and post-translational levels are poorly understood. Fujigaki et al. demonstrated that IDO activity is regulated by post-translational modification: specifically, IDO activity is inhibited by peroxynitrite due to the nitration of tyrosine resides (Tyr15, Tyr345, and Tyr353) (75). The same group also demonstrated that an N-terminal alanine of IDO is acetylated in IFN-γ-stimulated THP-1 cells, but the biological significance of this modification has not been fully investigated (76, 77). In addition, another group found that Tyr115 and Tyr253 in mouse IDO can be phosphorylated, and these phosphorylations are required for formation of the IDO/SOCS3 complex (78). This complex is ubiquitinated and subsequent proteasomally degraded in DCs. These studies indicate that post-translational modifications such as nitration and phosphorylation of tyrosine in IDO may affect IDO/kynurenine-mediated immune regulation. Recently, we showed that the miR-132/212 cluster participates in AHR-dependent generation of Th17 cells (79). miRs, 20–22-nucleotide non-coding RNAs, are a new class of regulators of gene expression at the posttranscriptional level. miRs binding to the 3′UTR of target mRNAs, leading to translational inhibition and/or degradation of the targets (80). Although numerous immunoregulatory genes are controlled by miRs, to date no miRs targeting IDO have been identified. According to a widely used computational miR target prediction tool, microRNA.org, several miRs potentially regulate IDO mRNA (http://www.microrna.org/microrna/home.do). For example, miR-203 has a putative binding site in the 3′UTR of IDO in mouse. Interestingly, this miR is induced by AHR ligands such as TCDD and BaP, and it negatively affects AHR expression in human cancer cell lines (81). In addition, it has been reported that miR-203 in macrophage RAW264.7 cells negatively regulates LPS-induced IL-6 and TNF-α by targeting MyD88 (82). Considering that AHR inhibits pro-inflammatory cytokines production such as IL-6 and TNF-α in LPS-stimulated macrophages as similarly to miR-203, miR-203 may also be involved in AHR-mediated regulation of inflammatory responses in macrophages. Although experimental verification is necessary in order to confirm this possible relationship between miR and IDO mRNA, identification of miR-mediated regulation of the IDO pathway may shed light on novel mechanisms of immune regulation.

## AHR/IDO Axis in Pathogenesis in Autoimmune Disease

**Figure 1:**
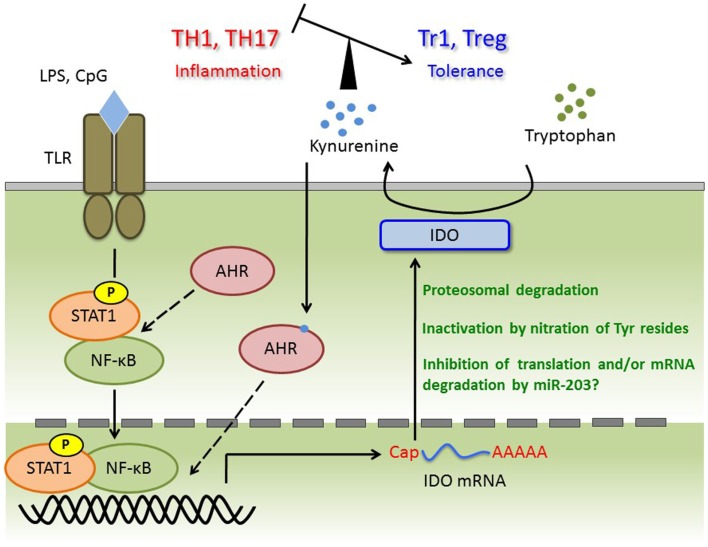
**TLR ligands trigger transcriptional activation of STAT-1 and NF-κB, and then induce IDO mRNA**. Although AHR forms complex with STAT-1 and NF-κB in macrophages under pro-inflammatory cytokines production, whether this complex is appeared in DC or required for IDO expression is not known. Induced IDO mRNA may be controlled by miR-203 (not investigated), and the activity or amount of IDO protein is regulated at post-translational modification such as nitration of Tyr and ubiquitin ligation. Kynurenine catalyzed by IDO induces tolerance via regulating the balance of TH1, TH17, Tr1, and Treg. Kynurenine may activate the AHR for IDO induction with autocrine manner, and form AHR/Kynurenine positive-feedback loop.

For several decades, AHR has been studied as an important transcription factor involved in regulation of a large superfamily of genes encoding cytochrome p450 proteins, which are xenobiotic metabolizing enzymes. Two independent groups demonstrated that AHR controls the generation of regulatory T (Treg) cells and/or IL-17-producing T helper (Th17) cells in EAE, a mouse model of MS (17, 41). Because the balance between Treg and Th17 cells is now considered to be more important than the Th1/Th2 balance in regard to the onset of autoimmunity, AHR has attracted increased attention in the context of immunology. To investigate the pathophysiological roles of AHR in autoimmunity, several groups have studied mouse models of autoimmune disease, such as colitis and rheumatoid arthritis, using AHR-KO mice and/or AHR ligands (83–85). Meanwhile, the roles of AHR in various immune cells such as DCs and macrophages have also been investigated (16, 25, 60). In DCs, AHR positively regulates IDO expression and subsequent kynurenine production (60). In addition, Kimura et al. observed reduced phosphorylation of STAT-1 in AHR-deficient macrophages; by contrast, AHR acts as a negative regulator of STAT-1 activation in T cells under Th17-polarizing conditions (15, 16). Thus, the effect of AHR on STAT-1 status is complex, and may be cell type- or stimulus-dependent. Given that expression of IDO is predominantly controlled by the IFN-γ/STAT-1 axis, AHR may positively control STAT-1 activation and subsequent IDO expression in DCs. More importantly, kynurenine is an agonist of AHR, and may participate in a positive-feedback loop in AHR signaling. In both plasma and CSF from MS patients, tryptophan levels were significantly reduced (86). In addition, IFN-β treatment, a first-line immunomodulatory treatment for MS, causes elevation of IDO mRNA and plasma or serum kynurenine (87, 88). As well as the observation in MS patients, EAE induction leads to alteration of the ratio of kynurenine and tryptophan or IDO expression in brain and spinal cord (89, 90). On the other hand, in spite of evidence that IDO is induced by IFN-γ, IDO expression is negatively correlated with IFN-γ mRNA levels in brain and spinal cord. This observation suggests that IDO activity inhibits the generation of IFN-γ-producing Th1 cells, a primary subset of pathogenic T cells (89). Consistent with these findings, deletion of IDO in mice and treatment with the IDO inhibitor 1-MT result in exacerbated disease symptoms in association with elevated levels of Th1 cells (89–91). We previously showed that LPS- or CpG-stimulated AHR-deficient BMDCs co-cultured with naïve T cells contain a smaller proportion of Treg cells and elevated levels of Th1 and Th17 (60). Furthermore, the aberrant generation of each Th-cell subset can be rescued by kynurenine. These observations indicate that kynurenine generates a tolerogenic condition by controlling not only Th1, but also Th17 and Treg cells. In addition, it has been reported that AHR participates in type 1 regulatory T (Tr1) cell generation *in vitro* and *in vivo* (18, 92, 93). Thus, we cannot exclude the possibility that not only TCDD and FICZ, but also kynurenine regulates Tr1 cell induction. Taken together, further investigation of the roles of kynurenine in T cell differentiation may reveal potential therapeutic strategies for MS. Thus, further investigation of the roles of kynurenine in T cell differentiation may reveal potential therapeutic strategies for MS. Immune regulation through the AHR/IDO axis is summarized in Figure 1.

## Perspectives of AHR/IDO Axis: From Bench to Bedside

Aryl hydrocarbon receptor and tryptophan metabolites participate in experimental models of autoimmune diseases (4, 17, 26, 41, 42, 61, 85, 94–96). However, the possible role of AHR in dioxin-exposed people is still unknown, particularly in autoimmune disorders. Therefore, it will be interesting to examine the expression of AHR and AHR-related genes in dioxin-affected people with the aim of identifying the potential link between AHR induction and dioxin-related autoimmune diseases. Future investigations should focus on determining whether activation of AHR leads to stimulation of IDO expression, and consequently promotes production of tryptophan metabolites such as kynurenine, which potentially mediate the neurological disorders and/or autoimmune diseases in dioxin-exposed people. Promising therapeutics based on intervention in the AHR/IDO axis may help to improve the health outcomes of dioxin exposure.

## Conflict of Interest Statement

The authors declare that the research was conducted in the absence of any commercial or financial relationships that could be construed as a potential conflict of interest.
